# That Criticism: (Dr. Briggs’ Reply to Dr. Wallace)

**Published:** 1887-02

**Authors:** J. R. Briggs

**Affiliations:** Dallas, Texas


### CORRESPONDENCE.

### THAT CRITICISM.

[DR. BRIGGS' REPLY TO DR. WALLACE'S REVIEW OF THE PRIZE ESSAY.]

Communicated for Daniel's Texas Medical Journal.

IN the December number (1886) of DANIEL'S TEXAS MEDICAL JOURNAL will be found an alleged criticism, by Dr. D. R. Wallace, of Terrell, of an essay by myself, to which was awarded the prize by the State Medical Association at its last meeting.

I have no objections to honest, legitimate criticism. In fact I rather court it, for when it is healthful—and all honest criticism is healthful—it is beneficial to myself as well as to the public, and above all, to the cause sought to be served.

Preliminary to the brief notice I shall give this alleged criticism, (I shall not dignify it with an elaborate reply) I desire to enter a respectful protest against the uncalled for and very seeming malicious attack upon the committee which awarded the prize in the contest of Essays at the last State Medical Association meeting.

Dr. Wallace says of the committee: "The gentlemen who made the award are honored members of an honorable profession. I have the profoundest respect for the personal character and professional status of all of them." Marc Anthony said Brutus was "an honorable man," yet Marc Anthony sought his overthrow and death. Rochefoucauld says "there is a sort of homage that vice pays to virtue." Will Dr. Wallace, who is so erudite, please state what sort of homage that is?

Was it necessary in the criticism of a Prize Essay to assail the committee, or rather the majority of the committee, which awarded the prize? Dr. Wallace was of that committee himself, but says he never saw the Prize Essay until November 15th, 1886, when "The Transactions" were published. Is not this fact the inspiration of his attack on the committee (the majority) who awarded the prize? He did not see the Essay. The calcium light—no, the electric blaze of his ponderous intellect never coming in contact with the pages of the Essay, was it not a presumption indeed, for this majority to make an award? It is a fact, the gentlemen who made the award are stars in the firmament of medical science, "honored members of an honorable profession," and "I have the the profoundest respect for the personal character and professional status of all of them." but stars only shine by reflected light, a reflected light from the sun, and the sun never having shone upon the Essay, these stars could not reflect the light of this sun, ergo their act was one of darkness. Great is Allah, and Mahomet is his prophet!

The good doctor says: "My name stands for something, in, as out of the profession in Texas—not much—how little, no one is more conscious than myself, but whatever it is, it is the result of work from the ground up, and I do not propose to be elbowed into prostituting it." Yes, it is evident the good doctor has worked "from the ground," and let us hope, "up." He has worked "from the ground" and alas! a goodly deal of the "earth earthy" remains.

If these gentlemen of the committee who awarded the prize are, in the estimation of Dr. Wallace, "honored members of an honorable profession," as he says, and if he has "the profoundest respect for the personal character," to say nothing of "the professional status" of all of them, how could they, being a majority, prostitute his name, or elbow him "into prostituting it," by awarding the prize to an Essay which, in their judgment, merited it? Which horn of the dilemma will the good doctor take? But enough on this branch of the question. The animus of the doctor's arraignment of the committee must be so plain that the blind may run and read; and now let us look to his criticism, "so called."

As I before stated, in all that I have ever written or that I ever may write, I court honest and legitimate criticism. The scalpel of the critic is just as legitimate in criticism, when it is needed, as is the knife of the surgeon in some diseases. But the skillful surgeon who knows when and how to cut, be it ever so deep that he puts his knife, does no mangling, leaves no jagged, rough and ugly scars behind to mark the presence of the butcher. Legitimate criticism is inspired by the worthy motives of correcting errors, combatting false theories, exposing illogical conclusions and making the right and the truth to triumph, and legitimate criticism is always honest, fair and just. It does not garble, it does not argue from false premises—that is from mis-stated premises; it does not build up men of straw to combat, palming them off as real men—it fights no men in buckram. The criticism that is otherwise than this, is born of petty malice, spite and hate, and brought forth in imbecile, puerile, pitiful envy or wounded self conceit.

Let us look at the animus of Dr. Wallace's self asserted criticism. Quoting from it he says: "In the first paragraph he [myself] says: It seems to me we, as physicians, are too prone to grasp gross facts rather than turn the channels of our investigations into the rich fields of forces and substantial though invisible entities which broad domain, with its protean manifestations are, [is] on every side, legion." I never said any such thing. He garbles what I said. He presents a man in buckram and proceeds to fight it with Falstaffian courage, skill and magnifying truthfulness. Before he gets through with the strife, there are seven men in buckram, and they have blown the doughty doctor fearfully in his bout with them.

What I did say is as follows: "It seems to me that we, as physicians, are too prone to grasp gross facts only, rather than turn the channels of our scientific investigations into the rich field of forces, and substantial, though invisible, entities, which broad domain, with its protean manifestations, are, upon every side, legion." I

give the words and punctuation just exactly as they appear in the "Transactions of the Texas State Medical Association" for 1886, page 545. Compare the exact words with the garbled paragraph of Dr. Wallace, and say if the animus of the criticism is not apparent. In the garbled paragraph in the alleged criticism, the word "that" is left out between the words "me" and "we," making it to read: "It seems to me we," instead of, "it seems to me that we etc. The elimination of the word "that" in this location is of small importance in itself, for I take it, it was really superfluous, but it was a mutilation. Let us look further. In the garbled paragraph, the word "only" is left out after the words "gross facts" and furthermore "gross facts" in the garbled extract are italicised, which is not the case in the essay. The elimination of the word "only," after the words "gross facts," very materially changes the sense of the sentence, and it is the sentence with the word "only" left out that Dr. Wallace assails. It is incumbent upon me to answer an attack upon something I never wrote? Must I poise a lance or cross swords with every Don Quixote, who, bestriding his Rosiante, and sighing for a Dulcinea del Toboso of literary renown, charges castles that live only in his disorded fancy?

But our modern Quixote, not content with the two mutilations already set forth, must needs indeed mutilate the paragraph a third time, and leave out the word "scientific" before the word "investitions." The force of this omission must be apparent to even the most casual reader. The critic must have been in sore straits indeed, for something against which to shape into words, "mere words," the drippings of his pen, when he has to mutilate a paragraph and distort it from its original reading, sense and meaning, to attack it.

It is the privilege of Dr. Wallace to believe, if he so desires, "that the whole catalogue of essences, quidities, entities, and whatnot constituting ontology, had been relegated to the owls and bats." He may quote Maudsley and Lord Bacon *ad libitum*, and be the apostle of these luminous geniuses, if he so wills, for that is none of my concern. He may raise his Maudsley to a pedestal in his own estimation as high as he pleases above St. Paul, he may canonize Lord Bacon above any of the old saints and martyrs, he may deny God, blaspheme Christ and raise altars to any strange god he pleases in the Athens of his intellect, and I will not gainsay his right, but I would like to know what this airing of his infidelity has to do with a legitimate criticism of the prize essay?

In the second paragraph of the essay the critic assumes to find matter worthy his attention. Here, as in the first paragraph we find him mutilating again. The paragraph in the essay reads: "Here is an inexorable quality of physical and mental law; not an abstract theoretical myth, but a veritable, vascular, live and active force, bequeating to our offspring certain physical, mental and moral traits of character not unlike ours." Dr. Wallace garbles it by leaving out the words "live and active" between "vascular" and "force," he makes the sentence, "here is an inexorable quality of physical and mental law" the text for an attempt at ridicule.

All who read the essay know the law referred to, is that law of inheritance by which, and through which the sins of the father are visited upon the children even to the third and fourth generations. Does Dr. Wallace deny the existence of such a law? Does he contend that man does not bequeath to his offspring the "physical, mental, and moral traits of character" he himself possesses? I say in the essay that such a law is "not an abstract theoretical myth, but a veritable vascular, live and active force." Does the critic claim that such a law is a myth, is "an abstract theoretical myth?" That it is not a "veritable" fact? That it is not a "vascular" force? That it is not "a live active force?" The force of his criticism of the paragraph in question is a total denial on his part of the law of inheritance in man and beast.

Dr. Wallace speaking of himself, says: "My name stands for something in and out of the profession in Texas." I am glad to know that it does. If his brow is decked with chaplets, it rejoices me; if his name is linked with fame, it gives me exceeding happiness. And let us wreath with the chaplet he wears, this other flower he hath won; *he denies the law of inheritance*; let us stud his coronet of fame with this other gem, all his own—*he denies the law of inheritance.* He demolishes my first paragraph with his infidelity and his creed, of the law of nature only, and he annihilates my second paragraph with a flat denial of the law of nature; and the inference of his optimistic belief that "there is a divinity that shapes our end," only.

The learned critic then jumps to another portion of the essay to misquote and garble as follows: "It is indeed surprising to find so little written upon rational principles pertaining to results accruing from hereditary predispositions beyond that of bare clinical facts, which if unformulated, are utterly worthless to the practitioner of medicine. In fact little is said in any way respecting hereditary disease—our standard text books devoting casual, indefinite attention to this subject." This is not what I said in the essay, but is another man of straw our doughty knight has set up at which to hurl his javelins. I said in the essay, and I will italicize the words omitted by the critic, as follows: "*In the study of this subject, we must say* it is indeed surprising to find so little written upon rational principles pertaining to results accruing from hereditary predispositions, beyond that of bare clinical facts, which if unformulated, are utterly *useless* to the practitioner of medicine. In fact *but* little is said in any way respecting hereditary diseases, our standard *medical* textbooks *merely* devoting casual *and* indefinite attention to the subject." It will be seen by a comparison of the two excerpts from the essay the exact one as I give it, and the garbled, mutilated one of the critic, that there is a very material difference in the two. Why did not the erudite gentleman criticise what I said? Why does he misquote me, thereby laying a false premise upon which to erect a very flimsy structure of assault? I say that "in the study of this subject" that "it is indeed surprising to find so little written upon rational principles pertaining to results accruing from hereditary predispositions. I said it and I adhere to it. Not one of the authors, W. B. Carpenter, H. Maudsley, Dick Hark Tuke, Bucknell and Tuke, Echeverria, Blandford, Bastian, Hammond or Gray, or even the learned critic himself in his luminous, super-esthetical, highly poetical, in-love-with-himself-young-man character of insanity reports, has written on the subject of which I treat and which I discuss in my essay. The very names of the works he gives by these authors are evidence of this fact, to say nothing of their contents. I have them in my library. I have read them and enjoyed their theories snd speculations, (the critic's Asylum insane reports excepted) but I have found them "useless," (not "worthless" as the critic has me to say in his misquotation) in my studies of the subject I handle, and so does every other student of the same subject. One of the authors quoted by the critic, Tuke, in his treatise on Action and Reaction of Mind on Body; in his work on the "Influence of Mind on Body" does treat to some extent on one branch of the questiou I handly in my essay; but can the critic, even in the profundity of his learning, and the immensity of his research, show what or how a disquisition on Epilepsy can have to do with "Reflections on Physical and Mental Culture "which was the basis of my Prize Essay? Festus said to Paul, "much learning doth make thee mad," and some one else has said "a little learning is a dangerous thing"; and I fear me it is the latter Charybdis and not the former Scylla that has wrecked our critic. At this particular point of Dr. Wallace's alleged criticism, I deem it my duty to "render unto Cæsar the things that are Cæsar's." I here pause in the course of my remarks in answer to his attempted criticism of my essay, to pay tribute to his modesty. With awe, and with veneration, and with profound wonder, I contemplate the immensity of this most exalted virtue, displayed by the erudite critic. He says, and he says it humbly: "But it is not in the limit of the possible for one man's head, however capacious, to hold everything." I shall never cease being thankful to Dr. Wallace for this acknowledgement. The Texas State Medical Association, and every physician within the broad limits of our great State, owes a debt of gratitude to Dr. Wallace for this admission, which they cannot repay, though they should live centuries. Whereabouts in the realm of thought; at what height which the imagination may attain even in its wildest flight; in what bright and beautiful spot within the far reaching scope of fancy, can any man find the resting place for the idea, the semblance even of a half formed thought, that Dr. Wallace ever, for an instant, in all his life, even dreamed that he did not *know it all;* that his "head" did not hold "everything"?

Dr. Wallace says: "After the first few pages he deals in nothing but platitudes of what is wrong in society; but if he mentioned one evil that exists in society, that is not common property, or one measure of reform that is not mentioned in the books, even in current newspaper literature, I trust he will have the kindness to point it out." The word "common" as used in the sense used by the critic in the above quotation according to Webster, means: "Belonging to the public or to all mankind; serving for the use of all; general." I ask Dr. Wallace if he will acknowledge that "clinical experience plainly develops the absurdity of atheistic teaching," (and I repeat that it does) "while it also shows that man is any thing but an angel"? (and this I also repeat.) Will he acknowledge these dicta? If not, then I have mentioned one evil (yes, two of them) which he does not know. Again I state: "Being guided by our intellectual and moral qualities, in all the ways of life, it would seem rational that we seek to better the human race by elevating our intellectual and conscious nature." Not to do this is an evil. Will Dr. Wallace gainsay that? Has he striven during his life, and his boasted 35 years of reading, while he was elevating (or seeking to do so) his intellectual nature, to also elevate his conscious nature that the human race might be bettered? If so, why does he say "the whole catalogue of essences, quidities, entities and what not, constituting ontology, had been relegated to the owls and bats?"—or in other words, why does he strive to injure and hurt and debase the human race by his infidelity?

The next point attempted to be made by the profound critic, is when he again misquotes, making me says: "There have been two and distinct classes of thinkers upon the subject of man's environment from the first days of human records, one purely and solely materialistic in character, advocating the evolution of man from the lowest forms of inferior animals, the other enthusiastic expounders of a highly spiritualistic theory of man's transcendant height above everything else of an animal nature." In this quotation the critic has left out the word "different" between the words "two" and "and" in the first line, and he makes me say "lowest forms of inferior animals" when I said "lowest types of inferior animals." These omissions and changes of the original text of what I did say, make no material difference, and I only mention them to show the critic was not honest, fair or just, "only this and nothing more." The critic wants to know what I mean by "man's evironments." I mean exactly what I say. Webster gives the definition of "environment" to be "act of surrounding; state of being surrounded; that which environs or surrounds;" and environ means, "to involve; to envelop; to surround; to encompass" etc., and I mean by "environment" just what Mr. Webster deflnes the word to mean. But the critic says: "It is taken for granted the writer means origin." I do not mean "origin." Webster defines origin to mean "beginning; first existence; source; birth; commencement." The trouble with Dr. Wallace as a critic, is, that he takes too much for granted. His *ipse dixit* must hold against any one else's opinion, and if his *ipse dixit* is gainsaid, it is a matter of course with him, that the man who gainsays it is either an ignoramus, or an idiot.

Having for many years been at the head of Insane Institutions, Dr. Wallace knows of course, that many men labor under hallucinations. He, unfortunately has one, and it is a very unhappy phantasm. It, like any *ignis fatuus leads him into quagmires* and makes him appear very ridiculous. He gives no man living, but Dr. D. R. Wallace, credit for knowing or understanding anything. Were the critic correct in his assertion, his taking it for granted that I meant "origin" when I meant "environments," there then might be some little reason and rhyme in his attempt to ridicule what he is pleased to term my "third theory of man's origin." I have made no attempt to formulate a theory of man's origin, either from a "scriptural" or from a "purely scientific standpoint," because the question of "man's origin" does not enter into the purview of my essay, but "man's environment" does, and it is of his "environment" I speak; and so much for that portion of the learned physician's labored effort, which reminds me very much of the saw, "the King of France marched up a hill and then marched down again."

Now Dr. Wallace, in his effort at a criticism of the prize essay, as he himself admits, has examined or touched upon but "ten pages" out of "eighty-four" and those the first ten pages, the introductory remarks to the essay. The cream, the essence, the real subject matter of the essay he has ignored, he has passed by; he has left unnoticed, and has contented himself with only the preliminary remarks. This must be another evidence that the animus which inspired the attack, it *is* nothing else, for it is unworthy the name of criticism; was not a noble and pure one, with the view to combat errors and to throw the light of truth upon intangible and untenable theories that the right might prevail; but rather the ignoble one, born of pique, wounded self importance, and an inordinate vanity.

As before said, every passage quoted by the Doctor for dissection has been misquoted and garbled, and his attacks have been made upon men of straw, his conclusions drawn from false premises.

I hardly take it to be incumbent upon me to indite an elaborate defense of the Prize Essay. It has been published in the "Transactions" and it is there to speak for itself, and wherein it ought to be criticised, wherein any principle it may advocate, any theory advanced, any idea or thought it may promulgate ought to be combatted, let it be done, for the truth's sake, for the right's sake; but because a man may be erudite; because he may have won some distinction as the head of an "insane hospital," because he may be able to write tolerably fair English and may possess the ability to a remarkable degree, to display his pedantry, is that a just reason, or adequate cause, or equitable justification for him to assail an humble and modest production, (which three honorable, able, and learned gentlemen have distinguished by awarding it a prize) as bravado, as a hired assassin with a bludgeon, may assail an unsuspecting victim?

Give us honest, earnest, genteel criticism, and he who objects is unworthy to write, or to labor in the field of science and literature, but protect us from the literary gabblers, the critics with not more than sense enough to be clerk in a literary bank, the pen and ink butcher, the bloody school of surgeons, who cut off legs and arms because they may have victims before them on the table. Amputate the legs and the arms when it is necessary, but don't do it with a carving knife and a hand saw, or a butchers cleaver.

Dr. Wallace writes English, if not forcibly and with perspicuity, at least tolerably well, but he has the unhappy knack of making raids upon a not very accurate memory for his efforts at humor, and upon his not extremely brilliant imagination for his facts.

He makes the committee say they gave me the prize because I am "a clever fellow" and that "he lives here with us." This is another variation from the path of strict veracity. I did not live in Dallas previous to, or at the time the award was made.

Also, there was no meeting of the committee of award on the "1st of April," as Dr. Wallace states. These are some of the "facts" utilized by this modern Solomon in "pushing forward the car of progress." I prefer to always adhere to "gross facts" rather than to "gross" misrepresentations.

This answer is already taking greater length than the nature, force and worth of the alleged criticism warrants, but before concluding what I have to say, I desire briefly to refer to the critic's animadversions upon my style of composition. It is a fact that no man's style is correct, absolutely; and it is a further fact that every man's style is *sui generis*. As long as a writer is grammatically correct, the manner of his writing is mere matter of taste, and no two tastes agree, as to literary form and method. That my style has not the polish and elegance, and rythmical symmetry of the learned(?) critic's, is a concession I freely make. Neither has what I write any lingering echoes from the notes of sweeter melodies wrought by the deft fingers, and born of the brilliant minds that have worked before me. There are no garnered threads from the looms of others running through the woof and warp; no colors from other pallettes than my own, to blend and mingle, either in harmonious beauty, or in frightful ugliness.

There may be lack of commas, and semi-colons; and colons and periods may have been incongruously mixed; in fact the make-up of the essay, by the printers, may be inartistic in the extreme, but the thoughts set forth therein are my own, and legitimately arrived at, and the whole speaks for itself, whatever there is of good in it to live, despite adverse criticism born of pique, or extravagant laudation, the output of a too partial friendliness.

In closing his assault Dr. Wallace says, "before signing my name to this communication, I have to make this proposition to Dr. Briggs: Submit his paper to three recognized psychologists, (I care not who) say Hammond; Seguin, of New York; Parrish, of Burlington, N. J.; Everts, of Cincinnati; Jewell, of Chicago—others equally qualified can be chosen—he to select one, the President of the Association another, and the third by myself. If they decide his a Prize Essay I will pay him $100 additional; if not, he returns the $100 prize money to the treasury of the Texas State Medical Association."

Now although this proposition was a gross and glaring insult to the gentlemen of the committee who awarded my essay the prize, an outrage of every principle of common decency and of ordinary gentility, yet I accepted his proposition.

"A change came over the spirit of his dream" however, after sleeping and reflecting upon his proposition, and on January 31st inst., nearly two months after making it, he writes me a letter changing the character and terms of that proposition. He says in his proposition at the close of his alleged criticism that the committee of psychological experts are to decide simply whether or not it is "a Prize Essay." "If they decide his a Prize Essay, I will pay him

$100, etc." In his letter to me he says (to quote his exact language): "To these parties knowing nothing of either of us or of *any contest*(?) your paper to be submitted without *name*—your name at top of page being cut off—*for their decision as to whether worthy to be eonsidered a Prize Essay in the generally accepted sense of the words*." The italics are my own. This is quite a modification of his first proposition, and is a sure manifestation that he leaped without looking, in his first proposition, and later, tries to find a loophole of escape. However, I do not accept his modified proposition, or acquiesce in his modificaiion. In accepting his first proposition I desire to disclaim any discourtesy to the gentlemen of the committee who awarded my essay the prize—but they know this. My only desire is for the essay to stand or fall upon its own merits, and not upon the *ipse dixit* of any self constituted Jupiter Tonans. A committee of learned, able, conscientious, honorable, honest, scientific gentlemen have passed upon my essay with eight others and to mine they awarded the prize, and their intelligence and learning is sufficient guarantee to me that it will stand the crucial test of even Dr. Wallace's egotism.

Dr. Wallace has not quoted me correctly in a single instance, besides he has handled the facts in the case with a slackness of truth that is indeed wonderful.

If an honest, earnest scholar of recognized ability should attack the principles of my essay, instead of ridiculing a few sentences in the introductory remarks, I shall then feel called upon to defend them, otherwise I shall feel that the essay is secure in the possession of a generous profession, many of whom have applauded me for my modest effort.

The letter I subjoin and comments, closes my reply to the renowned modern Cæsar's criticism(?) and goes to show how highly it is esteemed by his professional brethren.

This letter is one only of about fifty now in my possession of like nature.

"I have read with *pain* and *mortification*, and a terrible *big* disgust, the so-called review of your Prize Essay, by that * * * [epithets omitted, ED.] of Terrell. The personalities are contemptible, and at the present writing, recalling all of my meetings with him, I do not remember *ever* to have heard him speak a *good word* of *any man*. My dear sir, I *assure* you there are *many* who do not *entertain* or *endorse* the views as expressed by the reviewer. I do hope in your answer you will exercise your *usual* caution and dignity. How would it do to review his *puny effort* as printed with the transactions?"

The above letter speaks for itself and needs no comments, except to say that the writer is one of the ripest scholars and ablest physicians in this state. J. R. BRIGGS, M. D.

DALLAS, TEXAS, February 11th, 1887.

